# Anti-Hypertensive Effects of Peptides Derived from Rice Bran Protein

**DOI:** 10.3390/nu12103060

**Published:** 2020-10-07

**Authors:** Naohisa Shobako, Kousaku Ohinata

**Affiliations:** Division of Food Science and Biotechnology, Graduate School of Agriculture, Kyoto University, Gokasho Uji, Kyoto 611-0011, Japan; ohinata.kousaku.3n@kyoto-u.ac.jp

**Keywords:** rice bran, peptide, anti-hypertension, ACE inhibitory activity

## Abstract

Hypertension is one of the major risk factors for arteriosclerosis. Anti-hypertensive peptides derived from animal proteins, such as milk, eggs and fish, are well studied. Anti-hypertensive peptides have also been identified from plant proteins such as soybeans. Rice bran, a byproduct of white rice polishing, is rich in protein and its high protein efficiency ratio is well known. This review discusses the anti-hypertensive peptides identified from rice bran protein and their mechanisms. In addition, we describe protease-digested rice bran from which functional peptides have not been isolated.

## 1. Introduction

It is said that Hippocrates stated, “Let food be thy medicine and medicine be thy food.” [1]. Over a thousand years later, in the eighteenth century, Antoine Laurent Lavoisier demonstrated that respiration is a form of slow combustion [2]. Lavoisier thought food was burned to sustain life and that heat was released as a result. However, the components of food, such as carbohydrates, lipids and proteins, were not recognized at that time. These three components were identified in the nineteenth century. Digestion and absorption of these nutrients were also identified in this era [3]. During the late nineteenth to the twentieth century, other nutrients were found, such as vital amino acids and vitamins. In 1882, Kanehiro Takaki reported that a vegetable- and protein-rich diet reduced the risk of beriberi and in 1910, Umetaro Suzuki also reported that oryzanine (vitamin B_1_) extracted from rice bran cured beriberi [4,5]. Nutritional science in this era revealed the relationship between the nutrient components and life-related diseases, especially deficiency disorder. In the late twentieth century, the most concerning nutrient problem was the shift from starvation to satiation. Growing public awareness of the need to prevent metabolic syndromes, such as diabetes and hypertension, improved the quality of life. Peptides have attracted attention to overcome this problem. Peptides are short chains of amino acids connected by peptide bonds. Endogenous peptides exert unique physiological functions due to differences in amino acid sequences. For example, insulin was isolated by Frederick Banting and Charles Best in 1921, and its amino acid sequence was clarified by Frederick Sanger a few decades later [6,7]. Food-derived bioactive peptides were also isolated, such as β-casomorphin from casein peptone by Brantl et al., in 1979 [8]. Animal proteins, such as milk casein, fish protein and egg protein, were well studied as a source of bioactive peptides [9,10,11]. Plant proteins were also focused on as an origin of bioactive peptides such as anti-hypertensive peptides [12].

Hypertension is a key risk factor for cardiovascular disease, which affects one billion people worldwide. Control of blood pressure was a major issue until the 1940s. Franklin D. Roosevelt died from a hypertensive cerebral hemorrhage and his blood pressure was reported to be over 300 mmHg [13]. After 1950, anti-hypertensive drugs, such as α blockers, diuretics, calcium channel blockers and angiotensin I-converting enzyme (ACE, EC 3.4.15.1) inhibitors, were discovered [14,15]. ACE inhibitory peptides were first found from snake venom in the early 1970s [16,17]. ACE inhibitors from food proteins were first reported in 1979 by Oshima et al. [18]. Of note, this was the same year that opioid peptides derived from food were reported, as described above. ACE inhibitory peptides derived from food proteins have been used for foods for specific health use (FOSHU). Most of the ACE inhibitory peptides derived from food proteins are cleaved by protease digestion of protein-rich food material.

Rice *(Oryza sativa*), one of the major grains, serves as the staple food for almost half of the human population, and it is usually consumed in a polished form [19]. Rice bran, a byproduct of white rice processing, is rich in protein [20,21,22]. Approximately 10–20% of rice bran is protein, whereas endosperm contains only 6–8% protein [22]. The high protein efficiency ratio—defined as the ratio of protein that contributes to body growth [23]—of rice bran is well known [24]. As described above, rice bran protein is well studied but not well utilized. To reduce food waste, expanding the use of rice bran may be useful.

The objective of this review was to summarize anti-hypertensive peptides derived from rice bran protein. Methods for processing rice bran protein to exert anti-hypertensive effects were also summarized.

## 2. Anti-Hypertensive Peptides Isolated from Rice Bran Protein

### 2.1. Leu-Arg-Ala

In general, protein-rich food materials are digested by proteases or fermented by microorganisms, such as *Lactobacillales,* to produce anti-hypertensive peptides [25]. Several anti-hypertensive peptides are isolated from protease-digested rice bran.

**Figure 1:**
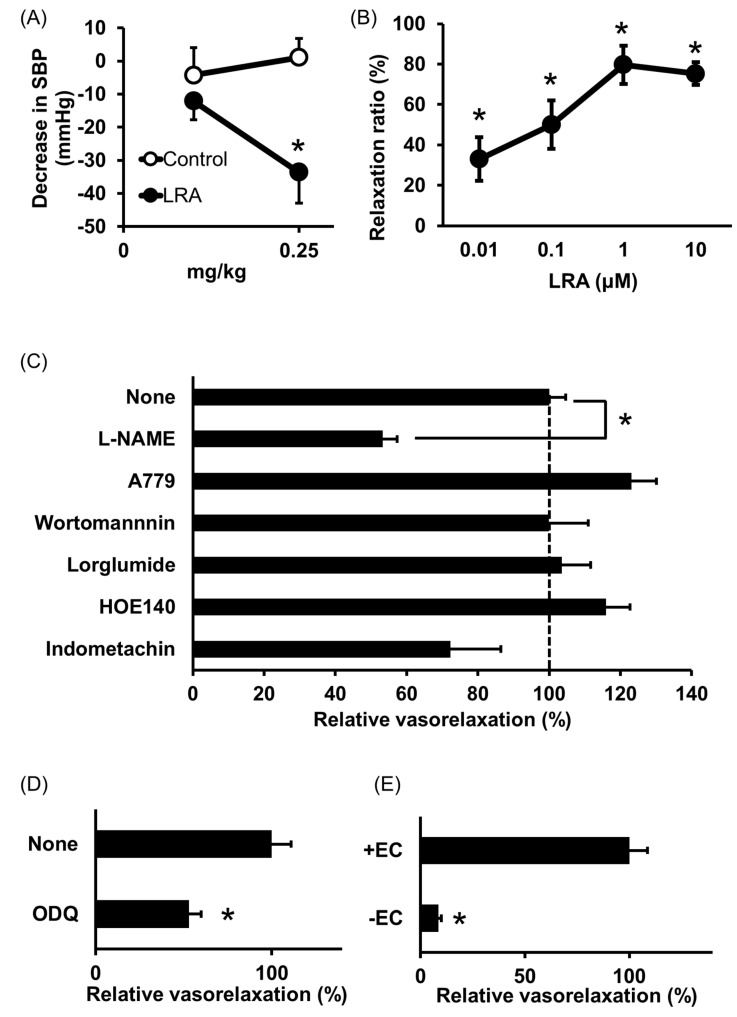
Anti-hypertensive effect of Leu-Arg-Ala (LRA). (**A**) Minimum effective dose was determined by in vivo study. Peptide samples were administered as a solution in saline. Each point represents the mean reduction in the systolic blood pressure (SBP) of SHRs and the vertical bars indicate the standard errors. * *p* < 0.05 indicates a significant difference compared with the control group, which was administered the same volume of saline (*N =* 5–10). These figures were modified and quoted from those previously reported by the author [26]. (**B**) Dose-dependency of the vasorelaxing activity of LRA. The peptide sample was applied for each concentration alone. The relaxation ratio was calculated using vasorelaxation with 100 μM papaverine as 100%. * *p* < 0.05, vs. water control group. (**C**,**D**) Effects of vasorelaxing pathway blockers that function in the endothelial layer (**C**) or vascular smooth muscle layer (**D**) on LRA-induced vasorelaxation. LRA = 10 μM, *N =* 4–8, * *p* < 0.05, vs. LRA alone. (**E**) Endothelial layer-removed samples (EC-) were also assessed. LRA = 10 μM, *N =* 3–9, * *p* < 0.05, vs. LRA alone. These figures were modified and quoted from those previously reported by the author [27]. SHR, Spontaneously hypertensive rats; L-NAME, N(G)-nitro-L-arginine methyl ester hydrochloride; ODQ, 1H-[1,2,4] oxadiazolo [4,3-a] quinoxalin-1-on.

Shobako identified the novel anti-hypertensive peptide Leu-Arg-Ala (LRA) from thermolysin-digested rice bran [26]. Its strong anti-hypertensive effects and vasodilating activity were previously reported [27]. Orally administered LRA demonstrated anti-hypertensive effects by Spontaneously Hypertensive Rat (SHR) examination and its minimal effective dose was 0.25 mg/kg (Figure 1A), which is the most potent anti-hypertensive peptide derived from rice protein.

**Figure 2:**
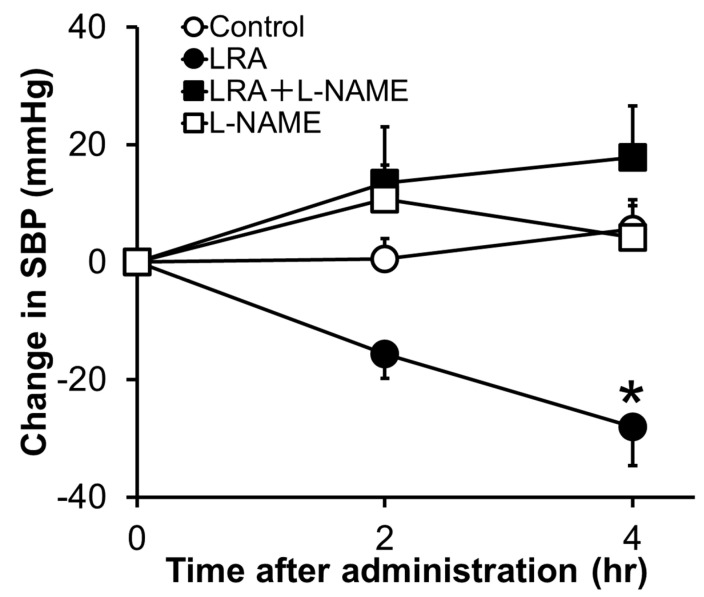
Effects of L-NAME, an NOS inhibitor, on the anti-hypertensive activity of LRA after oral administration in SHRs. L-NAME (20 mg/kg) was administrated just before the oral administration of LRA (1.0 mg/kg) or saline. The Y-axis represents the change in SBP from the beginning of the examination. Values are the mean ± SEM (N= 8–10). * *p* < 0.05, vs. control group. These figures were modified and quoted from those previously reported by the author [27]. L-NAME, N(G)-nitro-L-arginine methyl ester hydrochloride.

LRA exhibited potent vasorelaxing activity in the mesenteric artery isolated from SHRs, its half maximal effective concentration (EC_50_) value was 0.1 μM (Figure 1B); however, its ACE inhibitory activity was not as high (IC_50_ = 62 μM). EC_50_ values of food-derived vasorelaxant peptides, such as Arg-Ile-Tyr (Rapakinin) and Ile-His-Arg-Phe (IHRF), are 5.1 μM and 0.57 μM, respectively [28,29]. The vasodilating activity of LRA is the most potent among vasorelaxant peptides identified from grains to date. The vasorelaxing effects of LRA were inhibited by the nitric oxide synthase (NOS) inhibitor, N^G^-nitro-L-arginine methyl ester hydrochloride (L-NAME), and NO-sensitive guanylyl cyclase inhibitor, 1H-[1,2,4] oxadiazolo [4,3-a] quinoxaline-1-one (ODQ) (Figure 1C,D). Furthermore, vasodilation by LRA was not observed in the endothelial-removed mesenteric artery (Figure 1E) and its anti-hypertensive effects were inhibited by L-NAME in an in vivo study (Figure 2). These results suggest that an NO-mediated pathway is the main mechanism of the anti-hypertensive activity of LRA.

**Figure 3:**
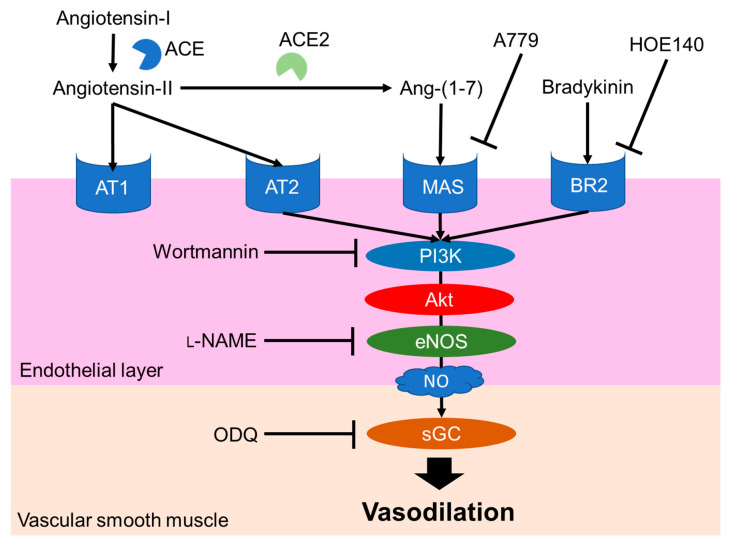
Major NO-mediated vasodilation pathways. Endogenous peptide hormones activate NO production by the phosphorylation of PI3K, Akt and eNOS. sGC, soluble guanylate cyclases; NO, nitric oxide; eNOS, endothelial nitric oxide synthase; Akt, protein kinase b; BR2, Bradykinin receptor B2, L-NAME, N(G)-nitro-L-arginine methyl ester hydrochloride.

The NO-mediated vasodilation pathway is well studied and a typical pathway is shown in Figure 3. Endogenous peptide hormones, such as angiotensin (1–7) and bradykinin, induce NO production through the PI3K/Akt/endothelial NOS (eNOS) pathway [30,31,32,33]. LRA also promotes eNOS phosphorylation, but LRA did not promote the phosphorylation of Akt in HUVEC cells, an endothelial cell model. The vasodilation activity of LRA was not inhibited by wortmannin (PI3K inhibitor) or HOE140 (BR2 inhibitor) [27]. Thus, factors upstream of NO production may be different from ang (1–7) and bradykinin. Other food-derived vasorelaxing peptides function via cholecystokinin (CCK) or prostaglandin I2 (PGI2) pathways, but LRA-induced vasorelaxation was not inhibited by lorglumide or indomethacin, a CCK antagonist and cyclooxygenase (COX) inhibitor, respectively [28,34]. Thus, LRA may relax the mesenteric artery via a novel pathway coupled to the NO system. This suggests that food-derived exogenous bioactive peptides, including LRA, and endogenous ligands can help reveal novel pathways in the cardiovascular system.

**Figure 4:**
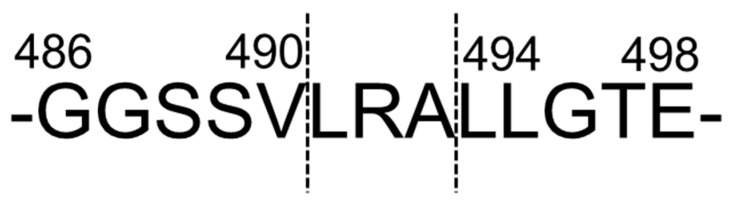
Predicted cleavage site of LRA from vicilin-like storage protein. These figures were modified and quoted from those previously reported by the author [26].

The origin of LRA was identified [26]; it was cleaved from a vicilin-like storage protein belonging to the cupin superfamily protein, one of the major rice bran proteins (Figure 4).

### 2.2. Tyr-Tyr

**Figure 5:**
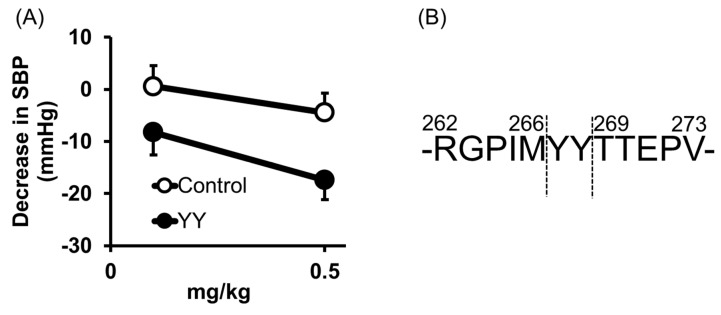
Anti-hypertensive effect of Tyr-Tyr (YY) (**A**) and predicted cleavage site of YY from vicilin-like storage protein (**B**). (**A**) Minimum effective dose was determined by in vivo study. Peptide samples were administered as a solution in saline. Each point represents the mean reduction in SBP of SHRs and vertical bars indicate the standard errors. * *p* < 0.05 indicates a significant difference compared with the control group, which was administered the same volume of saline (*N =* 5–10). (**B**) YY was cleaved from vicilin-like storage protein, at a different site from LRA. These figures were modified and quoted from those previously reported by the author [26]. SBP, systolic blood pressure; SHRs, spontaneously hypertensive rats.

Tyr-Tyr (YY) was also identified from thermolysin-digested rice bran as an anti-hypertensive peptide [26]; orally administered YY reduced the blood pressure at 0.5 mg/kg in SHRs (Figure 5A). Its high ACE inhibitory activity was also confirmed by an IC50 = 16 μM.

ACE is in a membrane-bound form in endothelial cells, neuroepithelial cells and the brain [35]. A soluble form was also reported, and is present in blood and different body fluids. ACE is a dipeptidyl carboxypeptidase that catalyzes the conversion of angiotensin I (Asp-Arg-Val-Tyr-Ile-His-Pro-Phe-His-Leu) to His-Leu and angiotensin II (Asp-Arg-Val-Tyr-Ile-His-Pro-Phe), a vasopressor peptide hormone [36]. ACE inhibitors reduce blood pressure by inhibiting ACE. ACE inhibition is a major mechanism of anti-hypertensive peptides derived from food-derived proteins. YY was also identified from protease-digested royal jelly and its high ACE inhibitory activity was previously reported [37].

In addition, the renin inhibitory activity of YY was previously reported [38]. Renin (EC 3.4.23.15) cleaves angiotensinogen to angiotensin-I and renin inhibitors are also used as anti-hypertensive drugs [39]. Renin inhibitory activity may lead to anti-hypertensive effects.

The origin of YY was previously identified [26]. It was cleaved from vicilin-like storage protein at a different site from LRA (Figure 5B).

### 2.3. Tyr-Ser-Lys

Tyr-Ser-Lys (YSK) was identified from trypsin-digested rice bran and its ACE inhibitory activity was measured as IC_50_ = 76 μM [40], being similar to LRA. The molecular docking study revealed that the ACE inhibition of YSK was mainly due to the formation of strong hydrogen bonds with the active pockets of human ACE.

It is well known that peptides exhibiting potent ACE inhibitory activity do not always exert strong anti-hypertensive effects [12]. An in vivo study of this peptide has not been reported and assessment of its anti-hypertensive effects in animal models is expected. However, there are many reports demonstrating that in vitro ACE inhibitory activity and in vivo anti-hypertensive effects are not linked. Further studies are also warranted to identify the cleavage site of this peptide.

### 2.4. Other Peptides

At present, no other anti-hypertensive peptides have been identified from rice bran. As described below, several reports demonstrated that protease-digested rice bran has anti-hypertensive activity, but its functional peptides are not well understood. Considering other effects, several other peptides were identified as functional peptides from rice bran. For example, VAGAEDAAK was isolated as an antioxidant peptide [20], LQPSHY had anti-melanogenic activity [41], and both IP and LP exhibited DPPIV inhibitory activity [42]. However, compared with animal-derived proteins, such as milk, egg and fish proteins, rice bran-derived bioactive peptides are limited. Further studies are expected to identify functional peptides from rice bran protein.

## 3. Anti-Hypertensive Effects of Protease-Digested Rice Bran

As described above, there are few anti-hypertensive peptides identified from rice bran protein. However, the anti-hypertensive effects of protease-digested rice bran have been reported even though functional peptides were not identified. In this section, the methods for producing anti-hypertensive food material from rice bran protein, regardless of whether the functional peptides were isolated, are summarized.

### 3.1. Thermolysin-Digested Rice Bran (TRB)

Thermolysin (*Bacillus thermoproteolyticus*) preferentially cleaves at the N-terminal side of hydrophobic or bulky amino sidechains such as Leu, Phe, Ile and Val [43]. Several thermolysin-digested food materials exhibited anti-hypertensive activity [44,45].

As described above, LRA and YY were identified from thermolysin-digested rice bran (TRB). Anti-hypertensive effects of TRB were demonstrated in an SHR study and human clinical study. A single oral administration of TRB reduced the systolic blood pressure (SBP) of SHRs at a dose of 30 mg/kg. Repeated oral administration for 4 weeks also reduced the SBP of SHR at a dose of 50–500 mg/kg/day. The administration of TRB at 500 mg/kg/day also reduced serum troponin I levels [26].

Administration of 1 g TRB (43 μg of LRA) for 12 weeks reduced the SBP in humans with high-normal blood pressure (SBP: 130–139 mmHg and/or diastolic blood pressure (DBP): 85–89) and grade 1 hypertension (SBP: 140–159 mmHg and/or DBP: 90–99 mmHg) [46]. This is the only study to demonstrate that rice bran-based food material alone can reduce blood pressure without being combined with another intervention. In this study, anti-hypertensive effects of TRB were also noted in the high-normal blood pressure subgroup. This suggests that TRB is useful for preventing the progression of pre-hypertension to grade 1 hypertension.

### 3.2. Protease G6-Digested Rice Bran

Protease G6, an alkaline serine endoprotease, is reported as a commercial proteolytic enzyme [47]. The anti-hypertensive effects of protease G6-digested rice bran (G6RB) were reported in in vivo studies and its mechanism was determined in previous in vitro studies. Boonla demonstrated the anti-hypertensive effects of G6RB in a rat model of two-kidney-one clip (2K-1C) renovascular hypertension, and promotion of eNOS expression in the thoracic aorta was also reported [48]. The anti-hypertensive activity of G6RB was also confirmed in Sprague-Dawley (SD rats fed a high-carbohydrate and high-fat (HCHF) diet and in L-NAME-administered SD rats. Upregulation of NOS expression was also observed in both studies [49,50]. The authors discussed that small peptides aid in the anti-hypertensive effects of G6RB, but they remain unidentified [50]. ACE inhibitory effects of G6RB were also observed in vivo [48]. Further studies on the isolation of anti-hypertensive peptides from G6RB and human clinical studies are warranted.

### 3.3. Trypsin-Digested Rice Bran

Anti-hypertensive effects of trypsin-digested rice bran (TRP) were previously demonstrated by Wang [40] using a single oral administration test on SHRs. It reduced the SBP and DBP of SHRs at a dose of 50 mg/kg. As described in Section 2.3, YSK was found from TRP. To confirm if this peptide is the functional substance of TPR, in vivo examinations are required.

### 3.4. Fermented Rice Bran

Fermentation is one method to produce anti-hypertensive peptides from food proteins. Anti-hypertensive peptides Ile-Pro-Pro (IPP) and Val-Pro-Pro (VPP) are cleaved from milk casein fermented by *Lactobacillus helveticus* and *Saccharomyces cerevisiae* [51]. The anti-hypertensive effects of rice bran fermented by *Aspergillus kawachii* and a mixture of lactic acid bacteria (*Lactobacillus brevis*, *Lactobacillus rhamnosus* and *Enterococcus faecium*) were demonstrated in an Stroke-Prone Spontaneously Hypertensive Rat (SHRSP) model and ACE inhibitory activity was observed in vivo [52]. Fermented rice bran (FRB) exhibited not only anti-hypertensive effects, but also improved glucose metabolism and the amount of triglyceride and total cholesterol in the liver [52]. Intake of FRB may increase plasma adiponectin levels, lead to the activation of AMPK, and downregulate gene expression related to glucose metabolism and lipid metabolism in the liver. It is possible that non-peptide components, such as adenosine [53] and ferulic acid [54], are the major contributors to the anti-hypertensive effects of FRB; therefore, the isolation of functional peptides is expected. Future human clinical studies are also expected.

This was an overview of the anti-hypertensive effects of processed rice bran and the summary is presented in Table 1. Many anti-hypertensive peptides derived from other food materials, such as milk, egg and fish proteins, were evaluated in human clinical trials. In particular, the anti-hypertensive effects of lactotripeptide derived from milk casein were well demonstrated in normotensive patients and those with high-normal blood pressure or grade I hypertension in several clinical trials [55,56,57]. The anti-hypertensive effects of TRB should be assessed in more trials, including in previously unexamined patient populations. Moreover, lactotripeptide was reported to improve endothelial function, usually measured by Flow Mediated Dilation (FMD) or plethysmography [34,58]. Endothelial dysfunction, which is also termed as the loss of arterial stiffness, is an important risk factor for cardiovascular events in hypertensive patients, and impaired NO bioactivity is thought to play a major role [59]. Thus, maintenance of the ability to produce NO in the endothelial layer is important for vascular function. Although the vasodilating effects of LRA—the functional substance of TRB—were similar to those of VPP in an ex vivo study [27], and the improvement of NO production was the key factor for the anti-hypertensive effects of G6RB, further clinical trials focused on endothelial function are required.

## 4. Conclusions

In conclusion, this review discussed the anti-hypertensive peptides derived from rice bran protein. LRA and YY were previously identified and predicted to originate from the same protein—vicilin-like storage protein. LRA exhibited strong vasodilating effects and promoted NO production in the endothelial layer. The mechanism underlying the anti-hypertensive effects of YY was reported as ACE inhibitory activity. Although the mechanisms of the anti-hypertensive pathway were different, these peptides demonstrated potent anti-hypertensive effects after oral administration. YSK was found from trypsin-digested rice bran and its ACE inhibitory activity was similar to that of YY. The anti-hypertensive effects and cleavage site of this peptide need to be clarified.

Crude processed rice bran, which was reported to have anti-hypertensive effects, was also reviewed. This is the first review especially dedicated to the anti-hypertensive effect of rice bran digestion. TRB was demonstrated to have anti-hypertensive effects in human clinical studies and anti-hypertensive peptides were identified as a functional substance. G6RB was also reported to possess anti-hypertensive effects and to promote eNOS expression in the endothelial layer. Furthermore, TRP exerts anti-hypertensive effects. Fermented rice bran has multiple effects other than anti-hypertensive effects, such as improving glucose tolerance and adiponectin production.

## Figures and Tables

**Table 1:** Summary of the anti-hypertensive effects of processed rice bran.

Process	Functional Peptide	Mechanisms	Animals/Human	Reference
Thermolysin digestion	LRA, YY	1.NO-mediated vasodilation 2.ACE inhibition	SHR model Human clinical trial	[26,27,46]
Protease G6 digestion	Not identified	1.Upregulation of NOS expression 2.ACE inhibition	SD rat (with L-NAME,2K1C) model	[47,48,49,50]
Trypsin digestion	YSK?	ACE inhibition	SHR model	[40]
Fermentation	Not identified	ACE inhibition	SHRSP model	[52,53,54]

LRA, Leu-Arg-Ala; YY, Tyr-Tyr; YSK, Tyr-SerLys; ACE, angiotensin-converting enzyme; NOS, nitric oxide synthase; SHR, spontaneously hypertensive rat; SD rat, Sprague-Dawley rat; L-NAME, N(G)-nitro-L-arginine methyl ester hydrochloride; 2K1C, two-kidney, one-clip; SHRSP, stroke-prone spontaneously hypertensive rat.
